# Water potential governs the effector specificity of the transcriptional regulator XylR of *Pseudomonas putida*

**DOI:** 10.1111/1462-2920.16342

**Published:** 2023-01-31

**Authors:** Pavel Dvořák, Teca Calcagno Galvão, Katharina Pflüger‐Grau, Alice M. Banks, Víctor de Lorenzo, Jose I. Jiménez

**Affiliations:** ^1^ Department of Experimental Biology (Section of Microbiology, Microbial Bioengineering Laboratory), Faculty of Science Masaryk University Brno Czech Republic; ^2^ Fiocruz, Instituto Oswaldo Cruz Rio de Janeiro Brazil; ^3^ Specialty Division for Systems Biotechnology Technische Universität München Garching Germany; ^4^ Department of Life Sciences Imperial College London London UK; ^5^ Systems Biology Department Centro Nacional de Biotecnología‐CSIC Madrid Spain

## Abstract

The biodegradative capacity of bacteria in their natural habitats is affected by water availability. In this work, we have examined the activity and effector specificity of the transcriptional regulator XylR of the TOL plasmid pWW0 of *Pseudomonas putida* mt‐2 for biodegradation of *m*‐xylene when external water potential was manipulated with polyethylene glycol PEG8000. By using non‐disruptive *luxCDEAB* reporter technology, we noticed that the promoter activated by XylR (*Pu*) restricted its activity and the regulator became more effector‐specific towards head TOL substrates when cells were grown under water subsaturation. Such a tight specificity brought about by water limitation was relaxed when intracellular osmotic stress was counteracted by the external addition of the compatible solute glycine betaine. With these facts in hand, XylR variants isolated earlier as effector‐specificity responders to the non‐substrate 1,2,4‐trichlorobenzene under high matric stress were re‐examined and found to be unaffected by water potential in vivo. All these phenomena could be ultimately explained as the result of water potential‐dependent conformational changes in the A domain of XylR and its effector‐binding pocket, as suggested by AlphaFold prediction of protein structures. The consequences of this scenario for the evolution of specificities in regulators and the emergence of catabolic pathways are discussed.

## INTRODUCTION

The physiology of bacteria in their natural niches is highly dependent on gross physicochemical conditions, among which water availability is a major player (Billi & Potts, 2002; Harris, 1981). The main factor to consider in this scenario is the so‐called water potential, for example, the effective potency of water in a given site compared to the pure counterpart. This parameter provides a measure (represented by the Greek letter Ψ and expressed in kilo or mega Pascals, kPa/MPa) of the relative tendency of water to move freely through a given space. Note that Ψ is never positive but has a maximum value of zero, that is, that of pure water. Ψ figures result from merging various environmental influences, including the presence of solutes, gravity, mechanical pressure, matrix effects, surface tension and others. Out of these, the main component of Ψ in the soil is matric potential (Ψ*_m_*), which results from the adhesion of water molecules to non‐dissolved structures of the environmental scenario at stake. These include the inorganic soil matrix, biomass, insoluble humic substances and any other type of soil particles. The rule of thumb is that the less water availability, the more negative Ψ becomes since any interacting molecules (whether dissolved or not) will restrict water's freedom to move. Due to the material complexity of soil structure and the pervasiveness of water subsaturation, it comes as no surprise that the matric potential of typical terrestrial niches critically frames microbial activity (Brown, 1990; Harris, 1981). This is because water is the vehicle for the intracellular circulation of metabolites, the flow of nutrients, signals and vapours among the biological actors of the site (Papendick & Campbell, 1981). All microorganisms have their optimum water potential for growth which varies over a continuous and extremely wide window of values—which themselves change with temperature and physiological conditions (Potts, 1994; Stevenson et al., 2015). Yet, many bacterial species are known to grow optimally at or close to a water potential of −0.05 MPa (Paul & Clark, 1989).

Biodegradation of environmental pollutants (e.g., aromatic hydrocarbons) by soil bacteria, requires adjusting their physiology and metabolic activities not just to the presence or absence of the substrates but also to the prevailing physicochemical settings of the place. Not surprisingly, water potential has been shown to greatly influence the transcriptome of bacteria exposed to solute or matric stress, that is, to low water potential (Gulez et al., 2012). But intriguingly, the vast majority of molecular studies on the catabolism of such chemical pollutants (Dvořák et al., 2017; Wackett & Hershberger, 2001) have been made in liquid media, whether minimal (e.g., M9, ~350 mOsm) or rich (e.g., Lysogeny Broth [LB] ~250 mOsm; Rothe et al., 2012). These figures reflect scenarios of high‐water availability quite different from those habitually found in soil. Beyond general physiological effects, the specific question is therefore to what extent water access influences pollutant biodegradation. This is not just a theoretical quiz, but an important information for planning bioremediation interventions.

Despite the diversity of approaches that one can entertain for tackling this issue (Dechesne et al., 2008; Gulez et al., 2012), the genetic and enzymatic system for catabolism of toluene and *m*‐xylene (XYL) encoded by the TOL plasmid pWW0 of soil bacterium and plant root colonizer *Pseudomonas putida* mt‐2 (Greated et al., 2002) offers a prime experimental system to this end. This is because expression of the so‐called TOL route is ultimately controlled by the prokaryotic enhancer‐binding protein XylR (Ramos et al., 1997).

**FIGURE 1:**
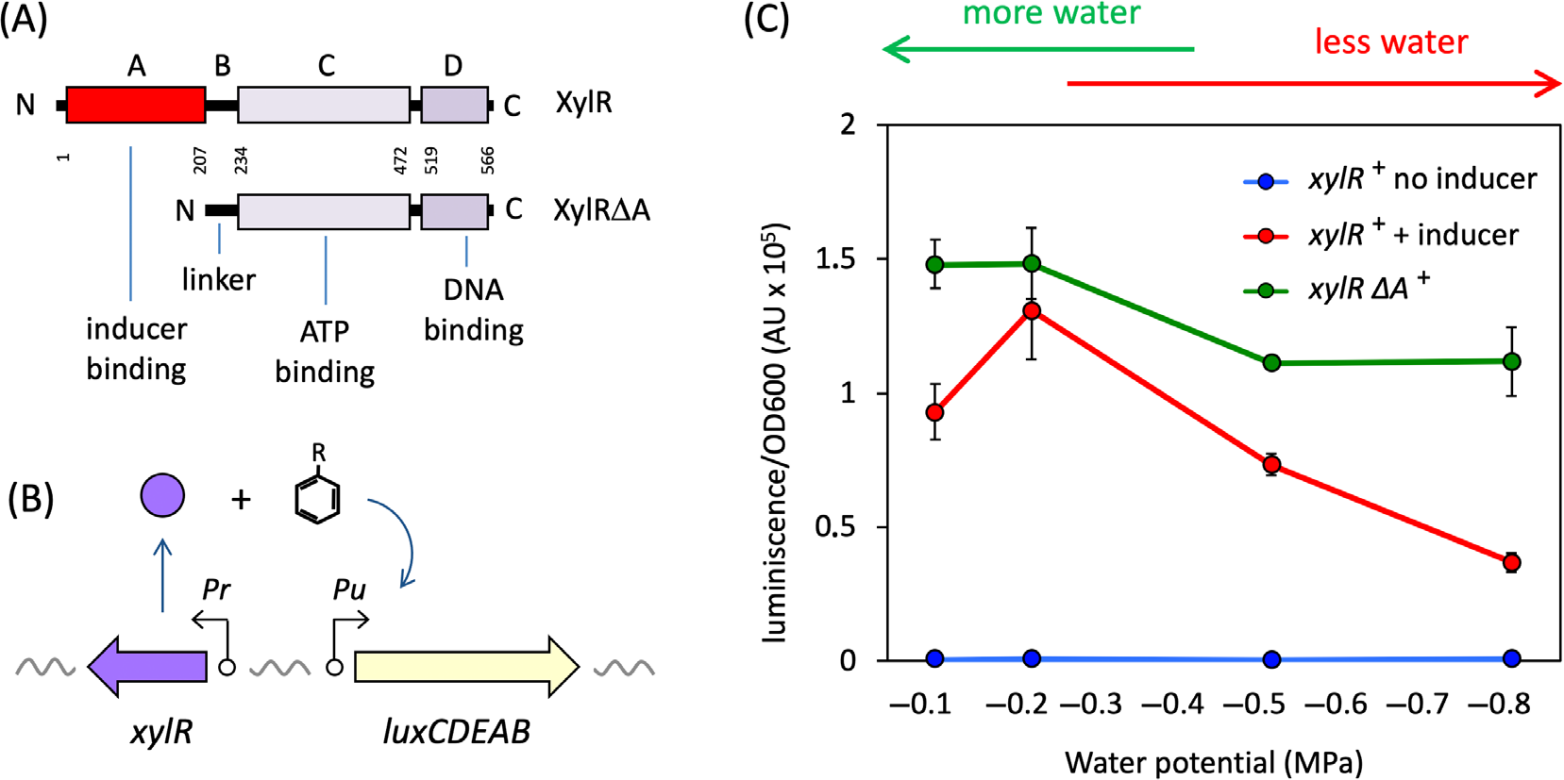
XylR response to matric stress depends on the A domain. (A) Domain organization of XylR. The sketches show the four domains of the regulator XylR and their main function together with their coordinates in the sequence of the protein represented as the first and last amino acids of each domain. A variant lacking the A domain, named XylR∆A, is not capable of binding to the inducer and it is always active. (B) Schematic representation of the XylR‐based biosensor. The strain contains two modules, one encoding XylR and the other one encoding a lux reporter for luminescence production under the control of target promoter *Pu*. When using the wild‐type XylR, the luminescence can only be detected in the presence of an inducer. However, when using the XylR∆A control, the production of the reporter is constitutive. (C) Response curves of the biosensors for different matric stresses. Water potential was tuned using different concentrations of PEG8000 and the production of luminescence was tested in each case. Red and blue lines represent, respectively, the response to matric stress of the strain *P. putida* CBPLUX (wild‐type XylR) in the presence of 1 mM 3‐methyl‐benzyl alcohol (3MBA) or the absence of an inducer. The blue line represents the response of *P. putida* MAD3 (XylR∆A) in the absence of the inducer. The data points show the mean ± standard deviation of four biological replicates (n.i. stands for not induced). For clarity negative values of water potentials are displayed in inverse order, being the lowest values (corresponding to larger amounts of PEG) on the right of the plot. We will follow the convention of increasing matric stress to the right in the rest of the figures.

Upon exposure of cells to either pathway substrates or close structural analogues, this factor activates the σ^54^‐dependent *Pu*, which turns on transcription of an upper *xyl* operon responsible for stepwise conversion of the aromatic hydrocarbons to the corresponding carboxylic acids (Ramos et al., 1997; Williams & Murray, 1974). XylR is organized into four modules with different functions (Figure 1A). The N‐terminal domain, named the A domain, is the sole responsible for inducer recognition (Fernández et al., 1995). This modular organization has been exploited to generate XylR‐variants with diverse effector specificities (Galvão et al., 2007) including a regulator lacking the A domain, which is constitutively active independently of the presence of an inducer (Fernández et al., 1995). Once benzoate or *m*‐toluate are generated, these intermediates are further channelled towards the central metabolism through the action of a separate lower pathway. The regulatory architecture of this system (Marqués & Ramos, 1993) and the domain structure of XylR enables examining separately the effect of any environmental condition on biodegradation proper, on the transcriptional machinery, and inducer recognition. An earlier phenomenological study (Holden et al., 1997) reported that while the growth efficiency of *P. putida* mt‐2 did not differ across the range of water potentials 0.0 to −1.5 MPa, toluene biodegradation was increased with a lower matric stress. But the molecular basis of such variability remains uncertain, as it is not related to either toluene transport or general physiological efficiency (Holden et al., 1997). This state of affairs suggests instead an effect of water availability on transcription of catabolic genes which then propagates into the biodegradative phenotype.

In this work, we have adopted non‐disruptive *luxCDEAB* reporter technology for inspecting the effect of water potential in the XylR/*Pu* regulatory node for degradation of aromatics by *P. putida* mt‐2 through induction of matric stress with polyethylene glycol 8000 (PEG8000). The genetic and biochemical evidence reported below unveils a thus far unknown role of water availability not just for checking expression of catabolic genes, but also for controlling inducer specificity of XylR by reshaping effector interaction with the A domain. This in turn has the potential to foster the evolution of new biodegradative phenotypes.

## EXPERIMENTAL PROCEDURES

### Bacterial strains and culture conditions

All bacterial strains used in this study are summarized in Table 1. *Escherichia coli* DH5α F' was used for cloning and plasmid propagation. *P. putida* KT2440 was used as the host for building biosensors. *E. coli* and *P. putida* were grown at 37°C or 30°C respectively in LB broth medium with vigorous shaking (170 rpm) and maintained using standard procedures. When needed, antibiotics ampicillin (Ap; 150 μg/mL), kanamycin (Km; 50 μg/mL), gentamycin (Gm; 10 μg/mL), or chloramphenicol (Cm; 30 μg/mL) were added to the culture media.

**TABLE 1:** Bacterial strains used in this study.

Strain	Characteristics	Source
*Escherichia coli* DH5α F′	*supE44* ∆(lacZYA‐argF)U169 ϕ80(l*acZ*∆M15) *hsdR17 recA1 endA1 gyrA96 thi1 relA1*	Invitrogen
*E. coli* CC118λpir	Cloning host: *araD139 Δ(ara‐leu)7697 ΔlacX74 galE galK phoA20 thi−1 rpsE rpoB* (Rif^R^) *argE(Am) recA1 λpir* phage	Herrero et al. (1990)
*E. coli* HB101 (pRK600)	Helper strain and plasmid for tri‐parental mating: F^−^ λ^−^ h*sdS20* (rB^−^ mB^−^) *recA13 leuB6* (Am) *araC14 Δ(gpt‐proA) 62 lacY1 galK2*(OC) *xyl−5 mtl−1 thiE1 rpsL20* (Sm^R^) *glnX44* (AS) pRK600 plasmid: oriV(ColE1) RK2 *tra* ^+^ *mob* ^+^, Cm^R^	Laboratory collection
*Pseudomonas putida* KT2440	Wild‐type strain, derived from *P. putida* mt‐2 cured of the catabolic TOL plasmid pWW0	Nelson et al. (2002)
*P. putida* CPLUX	Biosensor strain with wild‐type *xylR* inserted in *att*Tn7 site and *Pu‐luxCDABE* fusion introduced in chromosome using mini‐Tn*5* system (Gm^R^, Km^R^)	de las Heras and de Lorenzo (2011a)
*P. putida* MAD2	Control strain which harbours the ∆A‐version of *xylR* and a *Pu‐lacZ* reporter system in chromosome together with tellurite resistance gene	Fernández et al. (1995)
*P. putida* MAD3	Derivative of MAD2 strain in which *Pu‐lacZ* fusion was replaced by *Pu‐luxCDEAB* (Sm^R^)	This study
*P. putida* V101	Biosensor strain with *xylR* V101 variant (Y159F, L222R) inserted in *att*Tn7 site and *Pu‐luxCDABE* fusion introduced in chromosome using mini‐Tn*5* system (Gm^R^, Km^R^)	This study
*P. putida* V18	Biosensor strain with *xylR* V18 variant (S47R, L110S) inserted in *att*Tn7 site and *Pu‐luxCDABE* fusion in chromosome (Gm^R^, Km^R^)	This study
*P. putida* Va	Biosensor strain with *xylR* Va variant (M14I, I129N) inserted in *att*Tn7 site and *Pu‐luxCDABE* fusion in chromosome (Gm^R^, Km^R^)	This study

Abbreviations: Cm, chloramphenicol; Gm, gentamycin; Km, kanamycin; Sm, streptomycin.

We tested the response of the XylR wild‐type regulator using the *Pu‐lux*‐based reporter in *P. putida* CPLUX described elsewhere (de las Heras & de Lorenzo, 2011a). The control sensor that lacks the A domain of XylR and yields a constitutive output (*P. putida* MAD3) was built for this work using as a scaffold the strain *P. putida* MAD2 that harbours the ∆A‐version of XylR and a *Pu‐lacZ* reporter system (Fernández et al., 1995). The *Pu‐lacZ* fusion in that strain was replaced by *Pu‐luxCDEAB* from the plasmid patt PuLUX (de las Heras et al., 2011) as follows. First, the *Pu‐luxCDEAB* fusion was excised from the plasmid after XmaI and PstI digestion. The resulting fragment of 6158 bp was cloned into the likewise XmaI/PstI digested vector pBluescript SK(−) (Stratagene), where it was inserted at position 104 bp of *lacZα*. The resulting plasmid pBS_PuLux was subsequently digested with the restriction enzymes ApaI and SpeI, which led to a 6218 bp fragment. The restriction sites were chosen in a way to leave 54 bp of *lacZα* originating from pBS_PuLux fused to the 3′‐end of the *Pu‐luxCDEAB* fusion to allow homologous recombination with the *Pu‐lacZ* chromosomal insertion of *P. putida* MAD2. Next, this fragment was cloned into the *sacB* ^+^ suicide delivery vector pKNG101 (Kaniga et al., 1991) which was linearized with SmaI resulting in pKNG_PuLux. The correct insertion of the *Pu‐luxCDEAB* fragment into pKNG101 was confirmed by sequencing. Finally, the plasmid pKNG_PuLux was then mobilized from its specialized host *E. coli* CC118λpir (Herrero et al., 1990) into *P. putida* MAD2 through a triparental mating using *E. coli* HB101 (pRK600) as a helper strain (Martinez‐Garcia et al., 2011). Co‐integration of pKNG_PuLux into the chromosome was selected by plating the mating mixture on streptomycin (250 μg/mL) plates. To resolve the co‐integrates individual light‐emitting colonies (visualized with VersaDoc imaging system; Bio‐Rad) were grown in non‐selective LB medium overnight at 30°C and then plated on LB agar plates with 8% sucrose (w/v) to select for the second recombination event that released the plasmid from the chromosome. Correct replacement of the *Pu‐lacZ* fusion by the *Pu‐luxCDEAB* fusion was selected by streptomycin sensitivity, light emission and white colony forming on LB‐XGal Agar plates and then verified by PCR using different combinations of the primers Pu1F, lux3R, Pr2R and trp2R as described in de las Heras et al. (2008).

Biosensor strains containing wild‐type and variants of XylR (variants Va, V18 and V101) were constructed in a similar way. First, 713 bp DNA fragments encoding the AB domains of variants of interest were isolated after digesting plasmids pURXVa, pURXV18 and pURXV101 (Galvão et al., 2007) with EcoRI and AvrII. The resulting fragments were cloned in the plasmid pBX (de las Heras & de Lorenzo, 2011a) using the same restriction endonucleases. This vector is particularly useful since it allows the exchange of A domains in the regulator that is under the control of its native promoter *Pr*. The new set of plasmids generated was then digested with NotI and the 2612 bp fragments generated were cloned into de delivery vector pTn7‐FRT (de las Heras et al., 2008) generating the series pTn7‐BX (wild type), pTn7‐Va, pTn7‐V18 and pTn7‐V101. These constructions were finally co‐mobilized and used to insert the new versions of XylR into the natural *att*Tn7 of *P. putida* with a specific orientation (de las Heras & de Lorenzo, 2011a; Lambertsen et al., 2004). In addition to the helper strain and the receiver, this mating is also required to co‐mobilize the plasmid pTNS1 (Choi et al., 2005) encoding the Tn7 transposase. The receiver strain contained mini‐Tn5‐based insertion in the chromosome encoding the reporter system *Pu‐lux* (de las Heras & de Lorenzo, 2011a). In this case, transconjugants were selected on M9 minimal media containing citrate (0.2%) as the sole carbon source and the appropriate antibiotic. The location and orientation of the chromosomal insertions were verified by PCR using the oligonucleotides NDoCAvrII and PpuglmS‐2R as described in de las Heras et al. (2012).

### Matric stress

Water limitation assays were carried out in flasks using 50 mL of LB cultures. Cells were grown in LB medium until they reached an OD_600_ of 1.0. At that point, PEG8000 was added at the desired concentration and the cultures were incubated for 30 min prior to the addition of the inducer. We used concentrations of 0, 25, 100 and 200 g/L PEG8000 to generate water potentials of −0.15, −0.25, −0.50 and −0.75 MPa, respectively (Harris, 1981). All inducers were added at a final concentration of 1 mM except for XYL, benzene, and 1,2,4‐trichlorobenzene (TCB), which were provided in the vapour phase. For the osmoprotection assay, 1 mM or 10 mM final concentrations of glycine betaine were added to the culture medium at the same time as PEG8000. In all tests, the absorbance at 600 nm and the luminescence of 200 μL aliquots in 96‐well plates (NUNC) were monitored for 2 h after induction in a Victor II 1420 Multilabel Counter (Perkin‐Elmer). Luminescence is reported after normalizing by the OD_600_. Selected biosensors (V101, V18, Va) were tested in batch desiccation assays (50 mL) performed in flasks as shown before.

Assays using filters exposed to different humidity levels were conducted at 30°C in a room with a controlled humidity of 30%. 6 mL of a fresh culture of OD_600_ 1.0 were passed through a 0.22 μm filter to retain the cells. Filters containing the cells were deposited on an empty Petri dish with the cells facing up. The Petri dishes were then incubated in sealed desiccators which were either empty (i.e., contained room humidity) or filled with water (i.e., humidity saturation) for 2 h, followed by luminescence quantification. Humidity level and temperature in the desiccators were monitored with a digital hygrometer and a thermometer. Induction by vapour exposure took place by placing an open tube with TCB in the desiccator. Luminescence in the filters was imaged with a Geldoc XR (BioRad) without illumination.

### Structural models

The structures of XylR A domain wild type and variants were predicted from 211 amino acid‐long protein sequences using AlphaFold v2.2.0 (Senior et al., 2020). AlphaFold has been recently ranked as a top server in Community Wide Experiment on the Critical Assessment of Techniques for Protein 3D Structure Prediction (CASP, http://www.predictioncenter.org) and was therefore considered the most accurate tool for this study. The structural model of the wild‐type XylR A domain prepared previously by molecular threading with the I‐TASSER server (Iterative Threading ASSEmbly Refinement; Yang & Zhang, 2015) was visualized in PyMOL 1.6.x (Schrödinger) and compared with the structure calculated by AlphaFold using Align command: align modelWT_iTasser, modelWT_AFold, cycles = 0, transform = 0. The binding pocket and the Access tunnel in XylR A domain PDB file prepared in PyMOL were predicted using CAVER web 1.1 (Stourac et al., 2019). The pocket with the highest druggability score was selected and its volume was calculated in the wild‐type XylR A domain and in variants V18, Va and V101. The calculation was repeated five times for each structure and the mean values of pocket volume are presented with standard deviations. The data were treated with a two‐tailed Student's *t*‐test in Microsoft Office Excel 2013 (Microsoft Corp., USA), and confidence intervals were calculated. Pocket residues suggested by CAVER and manually verified in the modelled structure of wild‐type XylR A domain (F93, G96, P97, Y100, V108, L110, V124, A126, W128, Y155, A156, Y159, G160, F170, I185) are used in this work. Tunnels and bottleneck residues were calculated by CAVER using default programme parameters (except for the minimum probe radius which was reduced from 0.9 to 0.8 Å in the case of V101 and Va variants).

### Statistical analyses

The number of experiments and their replicates are specified in figure legends. The mean values and corresponding standard deviations (SD) are presented. When appropriate, data were treated with a two‐tailed unpaired Student's *t*‐test in Microsoft Office Excel 2013 (Microsoft Corp., USA) or using the T‐Test Calculator (https://www.graphpad.com/quickcalcs/ttest1/?Format=SD) and confidence intervals were calculated for selected values to manifest a statistically significant difference in means between two experimental datasets.

## RESULTS AND DISCUSSION

### Effect of matric stress on the transcriptional activity of the XylR/Pu regulatory node

The experimental system to inspect the impact of water availability on expression of *xyl* genes for toluene and XYL catabolism involves first liquid growth media with given water potentials. One effective method to this end entails the addition of given concentrations of non‐permeating solutes such as the high‐molecular‐weight polymer PEG8000 (Michel, 1983). The second component is the pair of reporter strains named *P. putida* CPLUX and *P. putida* MAD3 (Table 1; Figure 1A). Both of them come from the pWW0‐less derivative of *P. putida* mt‐2 strain called *P. putida* KT2440 (Table 1) and therefore they cannot degrade TOL pathway substrates. Yet, *P. putida* CPLUX and *P. putida* MAD3 carry each a chromosomal insertion of a *Pu‐luxCDABE* fusion, which acts as a non‐disruptive reporter of activation of the upper TOL pathway and thus operates as a proxy of metabolic activity (Figure 1B). *P. putida* CPLUX bears also an additional genomic insertion of the whole‐length, intact *xylR* gene, while *P. putida* MAD3 carries a variant of the same transcriptional factor deleted from the sequence encoding the A domain (*xylR∆A*). The consequence is that *P. putida* CPLUX cells emit luminescence only upon exposure to XylR effectors, while *P. putida* MAD3 has *Pu* constitutively turned on, without any inducer addition. Such a difference between the two strains allows separating the effects of any possible environmental condition on either the generic machinery transcribing *Pu* (or else the performance of the *lux* reporter system) from those involving only the interplay between the A domain and its effectors.

On these bases, we run the experiment shown in Figure 1C. Each *P. putida* strain (whether *xylR* ^+^ or *xylR∆A* ^+^) was grown in LB medium up to the early exponential phase and then supplemented with PEG8000 resulting in specific water potentials (Harris, 1981). The concentrations of the polymer added to the cultures caused growth reductions between 20% and 50% (Figure S1) thereby securing that cells were experiencing stress caused by PEG8000 addition. Samples were then added or not with 1.0 mM of the soluble XylR inducer and TOL substrate 3‐methyl‐benzyl alcohol (3MBA; Abril et al., 1989) and luminescence was recorded 2 h later (effector addition had no significant effect on growth, not shown). Note that the strains lacked the TOL plasmid and therefore they miss the capability to degrade the inducer, which remains in the culture media through the procedure. Inspection of the results of Figure 1C exposed various features of interest related to the question at stake. First, *Pu* activation in the *xylR∆A* ^+^ strain was only minimally affected by water potential within the interval of PEG8000 concentrations used, indicating that water potential had only a minor impact on both the basal transcriptional machinery and the functioning of the reporter system. Second, *Pu* activity induced by 3MBA in the *xylR* (wt) strain generally decreased with growing matric stress, an occurrence which aligns well with the decline of biodegradation activity observed in the original pWW0 plasmid‐bearing *P. putida* mt‐2 (Holden et al., 1997). And third, there was an intriguing peak of *Pu* activity at water potential ~−0.25 MPa, preceded and followed by lower luminescence values. Taken together, these data (i) accredited the value of the reporter *Pu‐luxCDABE* fusion for examining the influence of water potential on expression of *xyl* genes, (ii) narrowed down the origin of any possible effect to the interplay of the A domain with the aromatic effectors and (iii) hinted at an implication of matric stress in the interplay between the A domain of XylR and its cognate effectors. This last possibility was examined in detail as explained next.

### Water access determines inducer specificity

**FIGURE 2:**
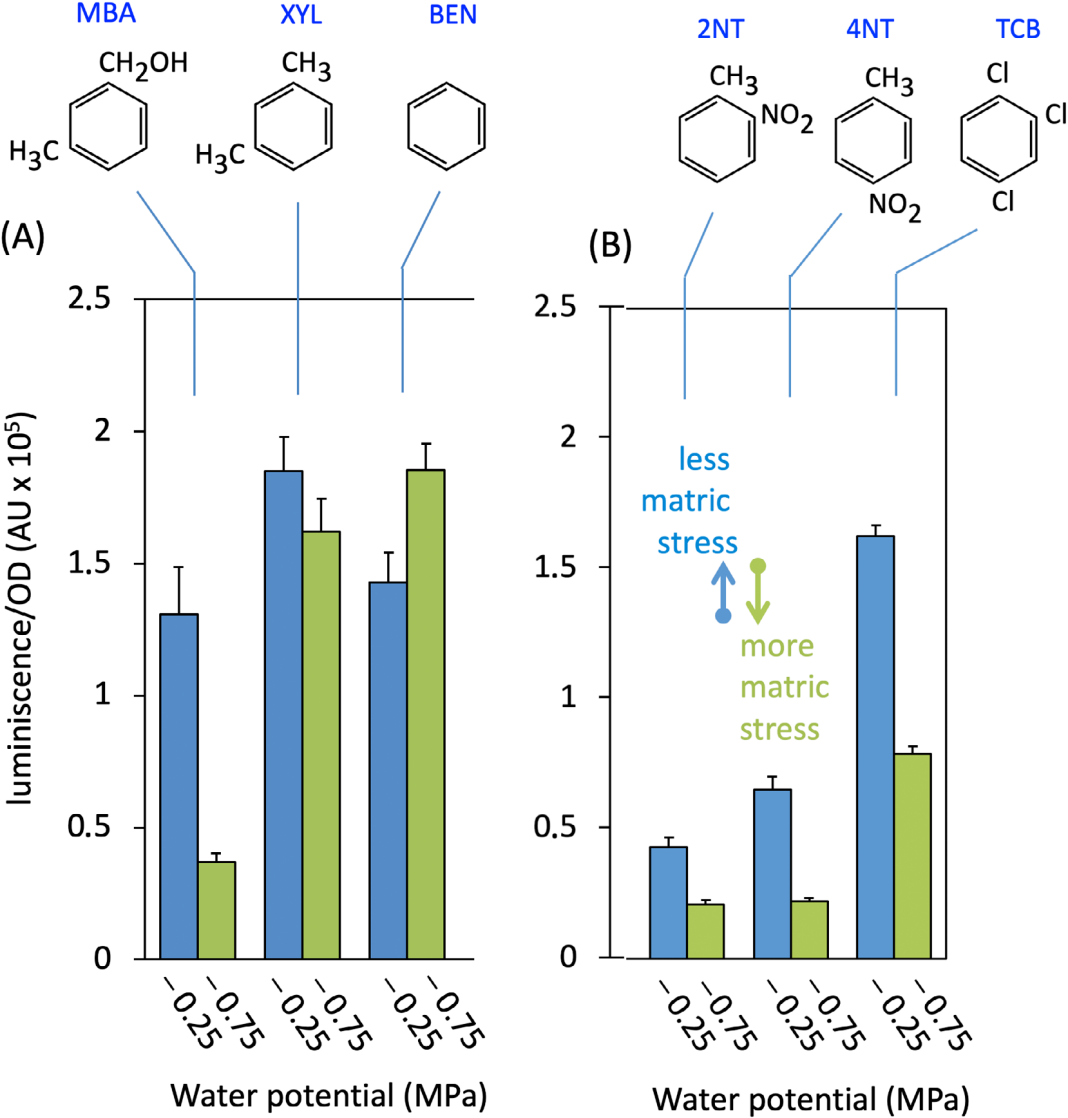
*Pu* activation profile by XylR varies with different inducers. Luminescence of the biosensor *P. putida* CPLUX was monitored in conditions of high (blue bars) and low (green bars) water potential in the presence of the following compounds: 3‐methyl‐benzyl alcohol (MBA), *m*‐xylene (XYL), benzene (BEN), 2‐nitrotoluene (2NT), 4‐nitrotoluene (4NT) and 1,2,4‐trichlorobenzene (TCB). MBA, 2NT and 4NT were added in solution to a final concentration of 1 mM whereas the others were used in the vapour phase. Columns show the mean ± standard deviation of three separate experiments. The changes in luminescence between the two conditions are significant (*p* < 0.01) for all compounds except for XYL and BEN.

Data of Figure 1C showed that the two apparent extremes of XylR‐mediated inducibility of *Pu* by TOL substrate MBA within the PEG8000 ranges tested happened at −0.25 MPa (lower matric stress, highest ON/OFF ratio) and −0.75 MPa (higher matric stress, lowest inducibility). These two values were thus adopted as a reference in the ensuing studies. The effect of matric stress on the response of XylR to the effector tested could be due to either change in the activity of the inducer‐bound regulator or to a poorer recognition of MBA as an agonist of the transcriptional factor. In reality, while 3MBA is an effective XylR inducer (Abril et al., 1989) its structure (Figure 2A) is significantly different from the head metabolic substrate of the TOL pathway (i.e., XYL). This is because 3MBA contains a bulkier polar alcohol side in the *meta* position occupied instead by an apolar methyl group in XYL. It is thus possible that the constellation of effector‐protein interactions evolutionarily optimized for XYL at the binding pocket of XylR may not be entirely equivalent for 3MBA. To examine this possibility, we compared the luminescence emitted by strain *P. putida* CPLUX (*xylR* ^+^, *Pu‐luxCDABE*) grown under a lighter or stronger matric stress when induced with either 3MBA or XYL. In addition, we tested under the same conditions the all‐apolar aromatic benzene as a potential XylR inducer as well. The results of Figure 2A show that the changes in the response of the XylR/*Pu* node to the suboptimal effector 3MBA with water potential were not kept when either XYL or benzene was used as inducers. These were minimally affected by matric stress.

The data of Figure 2A thus indicated that changes in environmental water availability kept unvarying the specificity of XylR for its authentic effector (XYL) while it modified the response to a non‐optimal inducer such as 3MBA. The result with benzene suggested that the phenomenon could affect inducers with polar groups while sparing all‐apolar mono‐aromatics. Should this be the case, suboptimal aromatic inducers (or no‐inducers) of approximate molecular size and shape as XYL but bearing polar groups could be upgraded to effective XylR effectors when cells were grown under low matric stress. To examine this prediction, we tested poor inducers (Galvão et al., 2007) 2‐nitrotoluene (2NT), 4‐nitrotoluene (4NT), and TCB and repeated the same *Pu* activity tests as before under lower (−0.75 MPa) or higher (−0.25 MPa) water potential. As shown in Figure 2B, it turned out that XylR‐mediated *Pu* inducibility by any of these compounds was significantly increased (*p* < 0.01) when cells were grown under low matric stress. This meant that productive interactions of XylR with otherwise non‐ideal inducers (including those well distant structurally from XYL, e.g., TCB) could be enhanced in media with an excess of water and thus XylR could become more effector‐promiscuous. In contrast, matric stress seemed to intensify the specificity of XylR for its bona fide effectors, that is, the head substrates of the TOL pathways. But what could be the mechanism behind this phenomenon?

### Endogenous osmotic stress tunes XylR effector specificity

**FIGURE 3:**
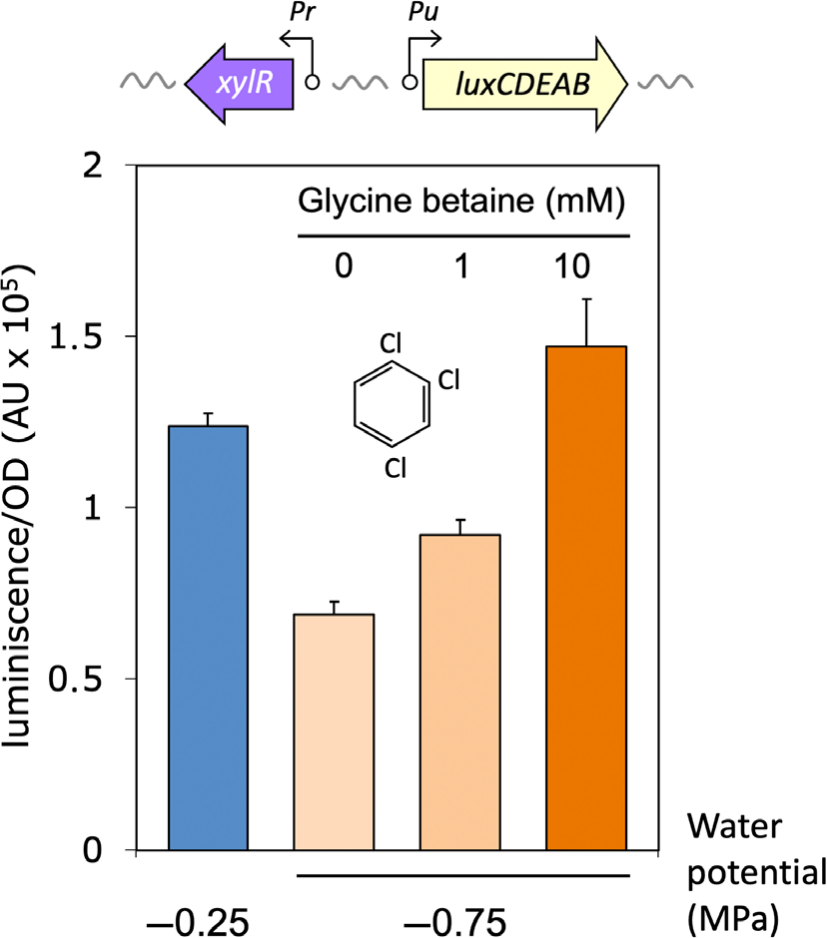
Restoration of *Pu* activation by the osmoprotectant glycine betaine in *P. putida* CPLUX. The figure shows the transcriptional activation of the *Pu* promoter by XylR in conditions of high (blue bar) and low (orange bars) water potential when induced with TCB. Upon the addition of the glycine betaine concentrations shown, the activation could be restored to normal levels. Columns show the mean ± standard deviation of three separate experiments. The changes in luminescence between betaine treatments are significant (*p* < 0.01).

The effect of water potential on XylR response to optimal and suboptimal inducers shown above could have a trivial explanation, for example, differences in transport of the externally‐added effector to its cytoplasmic target. Alternatively, the phenomenon could be traced to a different type of inducer‐regulator interaction due to changes in intracellular osmolarity brought about by changes in environmental water potential (Csonka & Hanson, 1991). Osmotic stress is generally met by bacteria by the production of compatible solutes (osmoprotectants; Csonka & Hanson, 1991). In particular, *P. putida* can accumulate the zwitterionic compatible solute glycine betaine either by synthesis or uptake (Galvão et al., 2006), as a way to compensate for a decrease in water potential. Should osmotic stress be at the basis of matric dependence of the XylR inducer profile, effector promiscuity in excess of water would then be restored if cells were treated with an externally added osmoprotectant. To test this prediction we picked the worst of all the potential inducers‐to‐be (TCB), which is generally considered either a very poor effector or no effector of XylR (Galvão et al., 2007). The ability of TCB to activate the *Pu‐luxCDABE* fusion of *xylR* ^+^ strain *P. putida* CPLUX was then followed at low (−0.25 MPa) or high (−0.75 MPa) matric stress in media exogenously amended with glycine betaine. The results shown in Figure 3 demonstrate how the effect of decreasing environmental water potential (and the ensuing intracellular osmotic stress) on TCB‐dependent and XylR‐dependent *Pu* activity was altogether suppressed when cells were provided with an excess of the compatible solute. These data added to the notion that water stress effects of the inducer‐specificity of the XylR/*Pu* node stem from intrinsic structural properties of the protein and the way its binding pocket recognizes the effector at different intracellular osmotic conditions. To further investigate this possibility, we adopted a genetic approach as described next.

### Revisiting XylR effector specificity variants responsive to TCB

Using the genetic and phenotypic selection methods described in Galvão et al. (2007), a number of variants in the A domain of XylR were isolated that had gained a new responsiveness to TCB which was by no means apparent in the wild‐type regulator. Examination of the protocol used indicated that selection for growth was made in agar plates, where colonies are subject to considerable matric stress (Hachicho et al., 2017), and thus—according to the data above—XylR effector specificity is expected to be the tightest. No wonder that under such conditions TCB behaves as a no‐inducer and variants that respond to the chloroaromatic compound could be duly interpreted as effector‐specificity variants. Yet, in light of the results above it could well happen that such TCB‐responsive variants just kept a native ability to respond to this compound which could now emerge by eliminating osmotic control. To inspect this possibility, we focused on XylR domain variants named Va (M14I, I129N), V18 (S47R, L110S) and V101 (Y159F, L222R) described in Galvão et al. (2007). Each of the corresponding sequences was inserted in the *att*Tn*7* site of a *Pu‐luxCDABE P. putida* variant to generate strains equivalent to *P. putida* CPLUX use above. This process (see Experimental procedures for details) rendered four isogenic reporter strains differing only by some amino acids in the A domain of the corresponding XylR variants.

**FIGURE 4:**
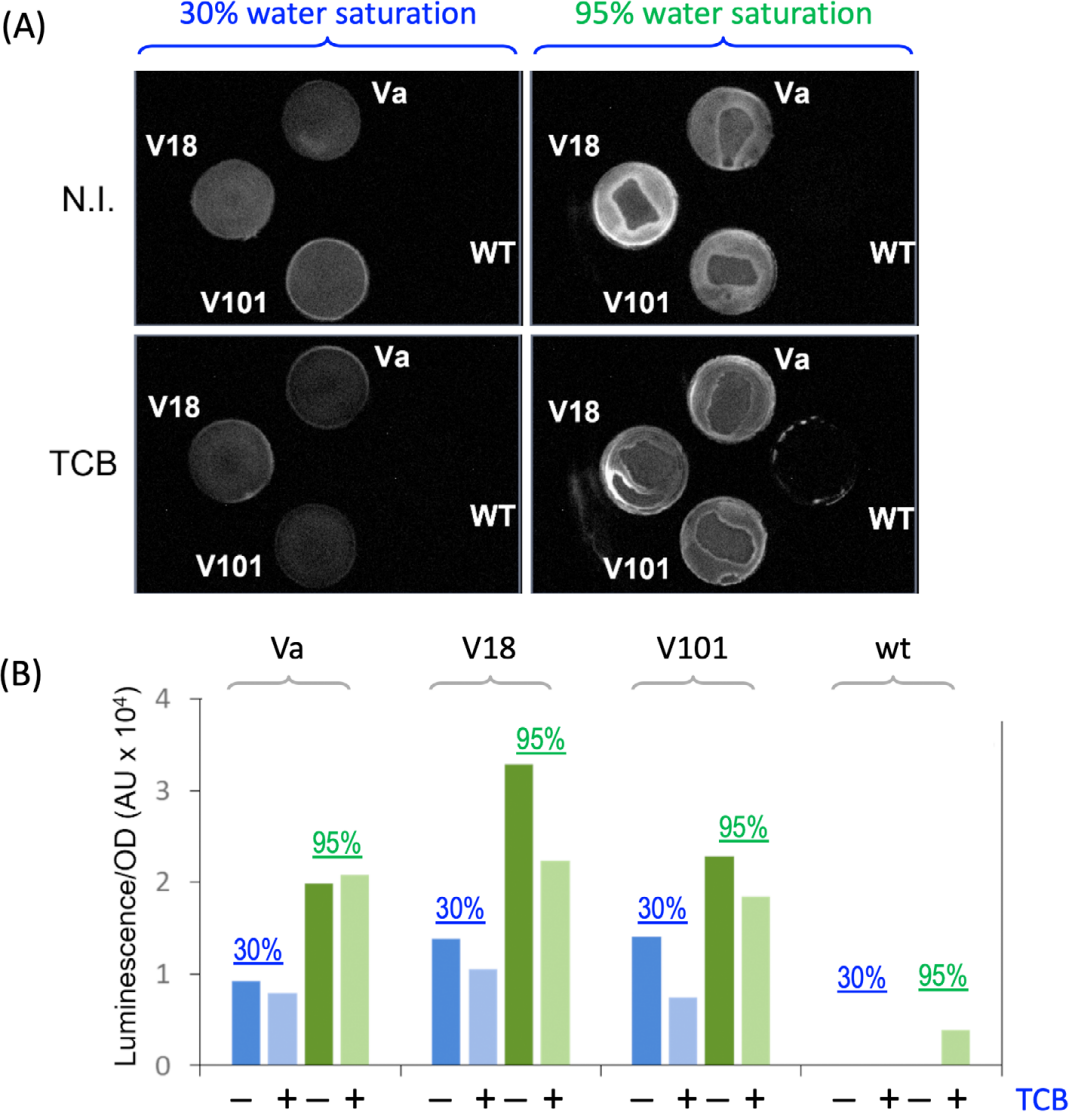
Activation of four biosensor strains with XylR A domain variants or wild type tested on 0.22 μm filters exposed to two different humidity levels. The figure shows the transcriptional activation of the *Pu* promoter in four *P. putida* biosensor strains with wild‐type XylR A domain or with A domain variants Va (M14I, I129N), V18 (S47R, L110S), or V101 (Y159F, L222R) on filters in low (30%, blue bars) or high (95%, green bars) humidity when induced or not with TCB as indicated. Variants of the XylR A domain gene were inserted in the *att*Tn7 site of a *P. putida* host with a *Pu‐lux* cassette.

To test the responsiveness of each of these XylR variants in a scenario of water limitation, each of the cognate strains was grown to OD_600_ 1.0 in LB medium and passed through 0.22 μm filters to retain the cells, placed (retained biomass face up) in an empty Petri dish, transferred to closed chambers with either 30% or 95% of controlled humidity (see Experimental procedures) and where indicated, exposed to saturating vapours of TCB. After 2 h, the luminescence emitted by each filter was imaged and quantified as shown in Figure 4. Although the information given by this experiment was more qualitative than quantitative, luminescence emitted by cells was clearly dependent on humidity. One important bit of information was that the wild‐type XylR could be activated to a noticeable degree of *Pu* output elicited by TCB under high‐water content but not with low humidity. Also, note that dependence on water is less pronounced in the strains with the regulator variants. Unlike wild‐type XylR, the other proteins were active both under low and high humidity which is consistent with the rest of the evidence pointing towards the A domain as a water sensor. In any case, the apparent levels of luminescence of all three *P. putida* strains bearing XylR variants were much higher at 95% than at 30% humidity regardless of the presence or absence of the TCB inducer. Therefore, Va, V18 and V101 XylR variants described before as new responders to TCB can be reinterpreted instead as proteins that have lost water control of XylR activity.

**FIGURE 5:**
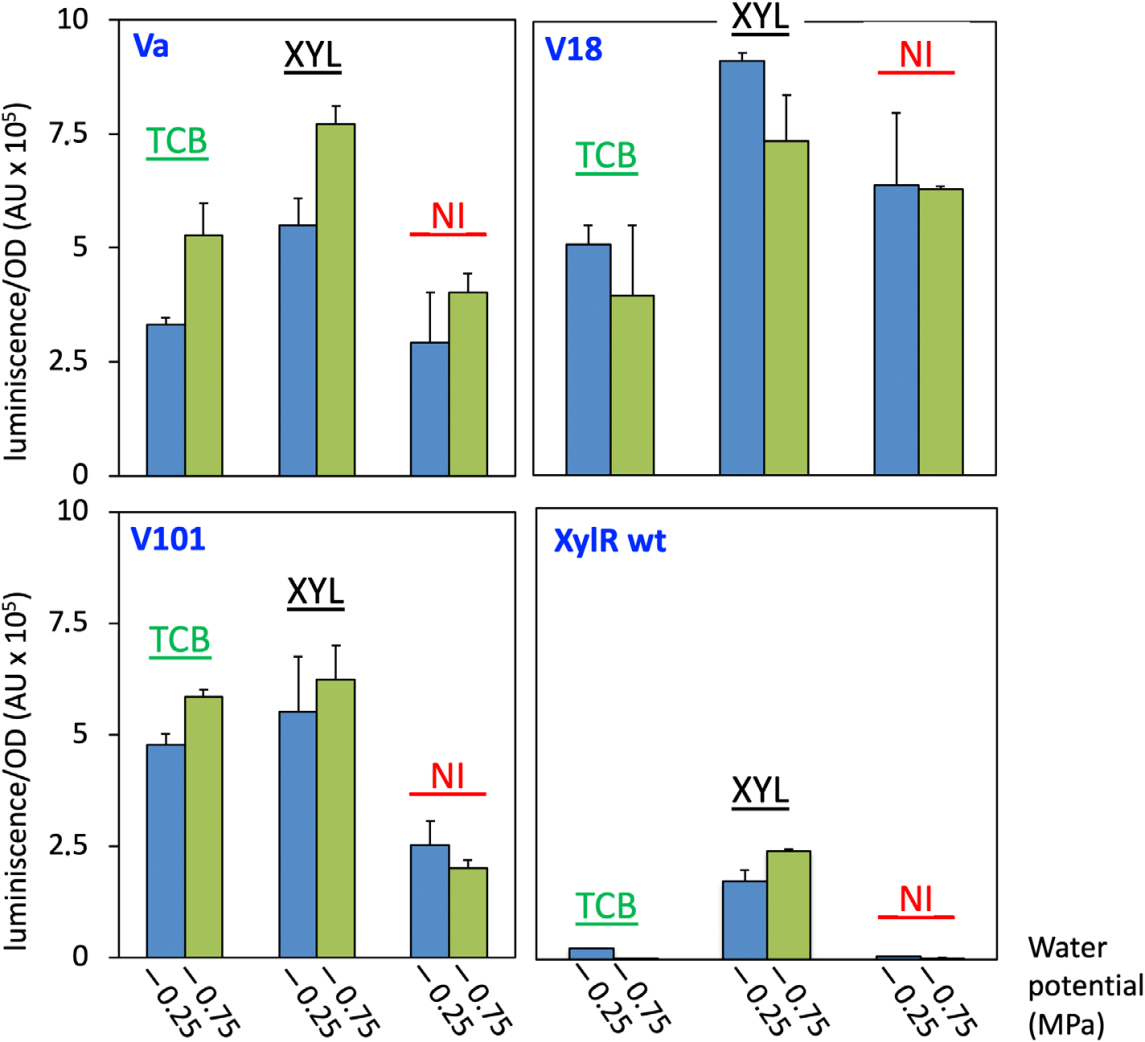
Molecular characterization of XylR response to matric stress. Different isogenic biosensors containing the variants Va, V18 and V101 as well as the wild‐type XylR were engineered and tested in desiccation experiments using high (blue) and low (green) water potentials and the inducers indicated (n.i. stands for not induced). Columns show the mean ± standard deviation of three separate experiments.

To gain further insight into this possibility we run quantitative water stress tests on the same variants grown in liquid media with PEG8000 as before when exposed to either TCB or the prime inducer XYL. The results displayed in Figure 5 show how the phenotype of TCB‐responsiveness, for which the variants were originally isolated in agar plates, unfolded in a variety of different behaviours when XylR‐mediated induction of *Pu* was tested under different environmental water availability conditions. As shown in Figure 5, the promoter activity of the XylR variants not only differed from the wild type after induction with TCB, but they also had a different pattern of *Pu* activity when induced with XYL. In general, the basal expression level of the variants in the absence of an effector was higher than that of the wild‐type regulator and they were less sensitive to changes in water potential (especially variants V18 and V101). Note that such minor sensitivity is in the order of that found in the control *P. putida* MAD3 harbouring the constitutive XylR∆A regulator (Figure 1C) and therefore the lack of response of the variants to water potential could indicate a complete release of any control by matric stress. Also, note that the V101 variant was less sensitive both to the prime inducer XYL and to the water potential than the wild‐type regulator. Not surprisingly, this particular variant was described in previous work as a *happy trigger*, or highly sensitive to induction (Galvão et al., 2007). Unexpectedly, the V18 variant was significantly inhibited—rather than activated—by TCB. Finally, variant Va displayed a noteworthy phenotype, as it responded better to either effector under matric stress (*p* < 0.01) than the wild‐type XylR when induced with XYL (*p* < 0.05). These data add to the notion that the performance of the A domain of XylR, in particular its ability to respond to non‐canonical inducers, depends on the water potential of the cytoplasmic milieu where the effector‐regulator recognition takes place. But is there a structural basis for this claim?

### The effector‐binding pocket of the XylR's A domain is prone to water‐dependent changes

**FIGURE 6:**
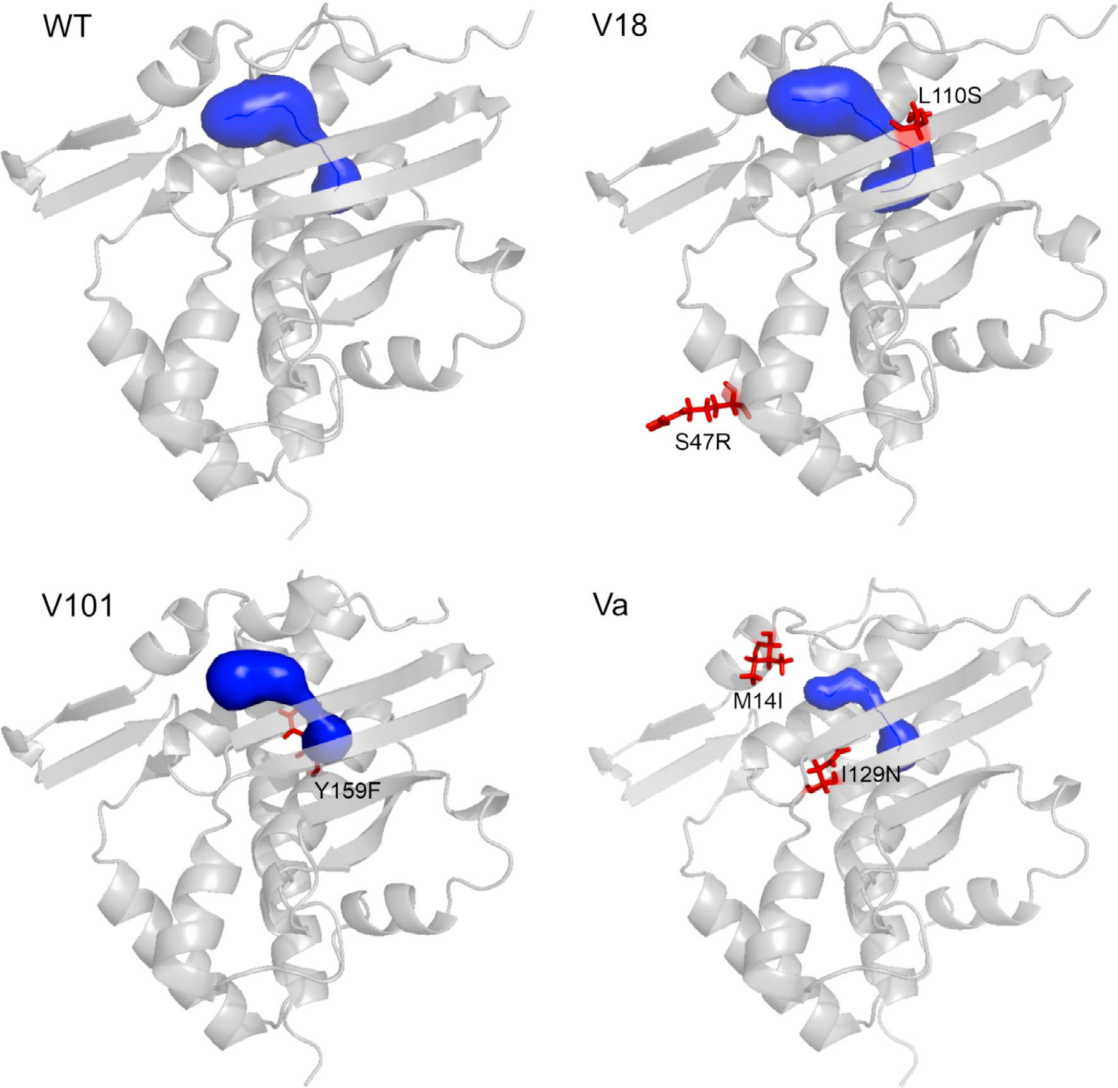
Distribution of mutations in the XylR A domain affecting the response to changes in water potential. Structures of wild‐type XylR A domain and variants V18, V101 and Va were calculated using AlphaFold and visualized using PyMOL. The binding pocket and entry tunnel in each of the structures were predicted by CAVER web 1.1 (Stourac et al., 2019). Structures are shown as grey cartoons, pockets with tunnels as blue surfaces, and mutations as red sticks. Note that L222R substitution is not included in the model of V101.

To make structural sense of the diverse genetic and phenotypic data above, the 3D model of the XylR A domain prepared previously by the I‐TASSER server (Dvořák et al., 2021) was further refined using the recently available AlphaFold structure prediction software (Senior et al., 2020). While the earlier and updated models differed only minimally (Figure S2), the predicted AlphaFold structure enabled an easy calculation and spatial location of the amino acid changes borne by the variants studied above. As shown in Figure 6, the modifications were located in the A domain pro, excepting L222R of variant V101, which is located in the Q linker between the A and C domains (Figure 1A). Changes in this region typically originate a semi‐constitutive phenotype (Garmendia & de Lorenzo, 2000) and they are believed to reflect alterations in the transmission of conformational changes to the rest of the protein after inducer binding to the A domain. V101 has an additional substitution in the highly conserved Y159 (Dvořák et al., 2021; Galvão et al., 2007). The Y159F variant lacks a hydrogen bond which probably stabilizes helices forming the bottom of the binding pocket and to which Y159 contributes (Dvořák et al., 2021). Y159F may thus cause the loosening of the binding pocket (Dvořák et al., 2021) and the combination of the two mutations in XylR V101 results in the high induction phenotype observed here and in the previous study of (Galvão et al., 2007). The rest of the substitutions (M14I, S47R, L110S, I129N) do not appear in residues conserved in the family of XylR‐like transcriptional regulators (Devos et al., 2002). One potentially revealing change (L110S) appears in the V18 variant (Figure 6 and Figure S3), as L110 was previously identified as one of the residues that narrow the entry tunnel connecting the bulk external solvent with the effector binding pocket of the protein. The other change borne by variant V18 is S47R in helix α2, a substructure that contributes to XylR dimerization (Dvořák et al., 2021). Finally, variant Va bears two amino acid changes. While M14I is very close to the entry of the newly calculated access tunnel and participates in its shaping (Figure 6 and Figure S3), I29N is in the vicinity of highly conserved residues that contribute to the hydrophobic core of the binding pocket (W128) and the dimerization interface (S131, F132 and E133). Importantly, the changes observed in the studied V18, V101 and Va variants seem to bring about a more expanded volume of the A domain tunnel and binding pocket (285.8 ± 8.2, 268.0 ± 6.2 and 237.2 ± 9.2 Å^3^, respectively) when compared with the wild type (225.0 ± 10.0 Å^3^). This expansion is statistically significant (*p* < 0.01) in V18 and V101 variants. Alterations in the binding pocket, differences in the channels to access the active site, modification of dimer maintenance and local variations in polarity caused by substitutions could affect the access of solutes to different regions of the protein. These could recreate in the variants the effect of water in an otherwise malleable, flexible architecture of native XylR. The only way to reach the active centre of the A domain (which is buried within the protein structure, Figure S3) is by having a very plastic conformation that can either expand for enabling the entry of the inducer‐enlarged channel or by aiding protein folding (Dvořák et al., 2021). Conformational changes in proteins caused by water availability are well known (Goldbeck et al., 2001; Harries & Rösgen, 2008; Levy & Onuchic, 2006) and the evidence presented in this work makes plausible that they are the basis of the phenomena reported above.

### Conclusion

The data presented in this work accredit that (i) the known effect of matric stress on the biodegradative ability of *P. putida* mt‐2 (Holden et al., 1997) aligns well with the performance of the XylR regulator under varying water potentials, (ii) inducer specificity of XylR‐controlled transcription from *Pu* narrows down to pathway substrates (e.g., XYL) at high matric stress (low water potential) but the same is relaxed under low matric stress (or addition of osmoprotectants) to include aromatics with considerably different structures and (iii) some variants in the A domain of XylR that make the regulator insensitive to water potential seem to make the protein adopt a structure more accessible to water and other solutes. The effect of water on the *Pu* promoter in vivo can thus be unequivocally traced to the protein itself and not to any other upstream (e.g., transport) or downstream (e.g., *Pu* functioning) steps of the transcription process. While the *P. putida* strains used in this work are not rigorously isogenic, their pair‐wise comparisons under the conditions tested consistently substantiate the claims put forward by this research.

In sum, the expression of the biodegradative *xyl* genes encoded in the TOL plasmid pWW0 under matric stress is mostly limited to bona fide substrates while the same can be induced by non‐metabolizable aromatics with some structural resemblance. In other words, the effector profile of XylR is tuned by environmental water availability, plausibly through water‐dependent changes in the access to the binding pocket and/or variation in the geometry of the interaction sphere. This is not without precedents as many proteins can change their conformation in a water availability‐dependent fashion (Levy & Onuchic, 2006). Furthermore, the structural effect of water molecules sitting in highly conserved positions within the same family of proteins can be recreated by site mutations in key residues of the polypeptide (Bottoms et al., 2002; Knight et al., 2009).

Water‐dependent tuning of promiscuity of regulators such as XylR may shed some light on the evolution of new specificities in catabolic pathways, which starts by co‐opting and amplifying minor responsiveness to a new effector (de las Heras & de Lorenzo, 2011b; de Lorenzo & Pérez‐Martín, 1996). In light of the data above, this occurrence might be more efficient in freshwater systems than in soil, at least for some bacterial species and pathways. Similarly to enzymes (Aharoni et al., 2005; Bloom & Arnold, 2009), specific recognition and adapted performance to environmental conditions may evolve from stem protein types with lower general fitness but a broader range of potential functions. Any condition that increases promiscuity (like water excess, as entertained above) could thus allow a faster evolution of transcriptional factors towards new specificities.

## AUTHOR CONTRIBUTIONS

**Pavel Dvořák:** Formal analysis (equal); investigation (equal); methodology (equal); software (equal); validation (equal); writing – original draft (equal); writing – review and editing (equal). **Teca Calcagno Galvão:** Investigation (supporting); methodology (supporting); validation (equal). **Katharina Pflüger‐Grau:** Investigation (supporting); methodology (supporting); validation (equal). **Alice M. Banks:** Investigation (supporting). **Victor de Lorenzo:** Conceptualization (equal); funding acquisition (equal); investigation (equal); resources (equal); writing – review and editing (equal). **Jose I. Jiménez:** Conceptualization (equal); investigation (equal); writing – original draft (equal).

## CONFLICT OF INTEREST STATEMENT

The authors declare no conflict of interest.

## ETHICS STATEMENT

The work presented in this article follows all prevailing local, national and international regulations and conventions, and normal scientific ethical practices.

## Supporting information


**FIGURE S1.** Growth of *Pseudomonas putida* KT2440 at various levels of matric stress elicited by addition of PEG8000 to cultures
**FIGURE S2.** Comparison of XylR A domain models prepared by I‐TASSER or AlphaFold.
**FIGURE S3.** Access tunnel, binding pocket and relevant amino acid residues that contribute to their shaping in the AlphaFold model of the wild‐type XylR A domain.

## Data Availability

The data that support the findings of this study are available from the corresponding author upon reasonable request.
