# Supplementary material for: Water potential governs the effector specificity of the transcriptional regulator XylR of Pseudomonas putida
Source: Environ Microbiol. 2023 Jan 31;25(5):1041–54. doi: 10.1111/1462-2920.16342 (PMC10946618; doi:10.1111/1462-2920.16342)
Supplement: Supplementary file 1 — FIGURE S1. Growth of Pseudomonas putida KT2440 at various levels of matric stress elicited by addition of PEG8000 to cultures FIGURE S2. Comparison of XylR A domain models prepared by I‐TASSER or AlphaFold. FIGURE S3. Access tunnel, binding pocket and relevant amino acid residues that contribute to their shaping in the AlphaFold model of the wild‐type XylR A domain. [file EMI-25-1041-s001.pdf]

Supplementary Figure S1. Growth of *Pseudomonas putida* KT2440 at various levels of matric stress elicited by addition of PEG8000 to cultures

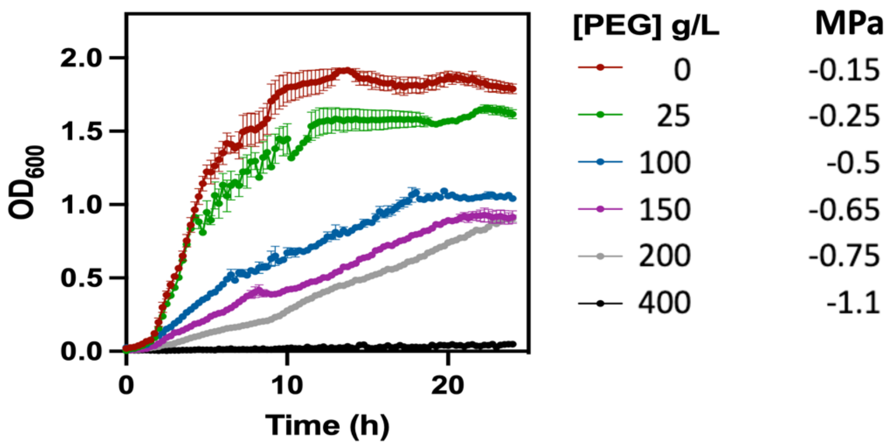

Triplicate cultures of *P. putida* KT2440 were grown in LB medium at 30ºC in the presence of the concentrations of PEG8000 indicated, and its OD_{600} recorded along time as shown. Note the inhibitory effect of the additions, which is virtually total at the highest PEG8000 addition.

Supplementary Figure S2. Comparison of XylR A domain models prepared by I-TASSER or AlphaFold.

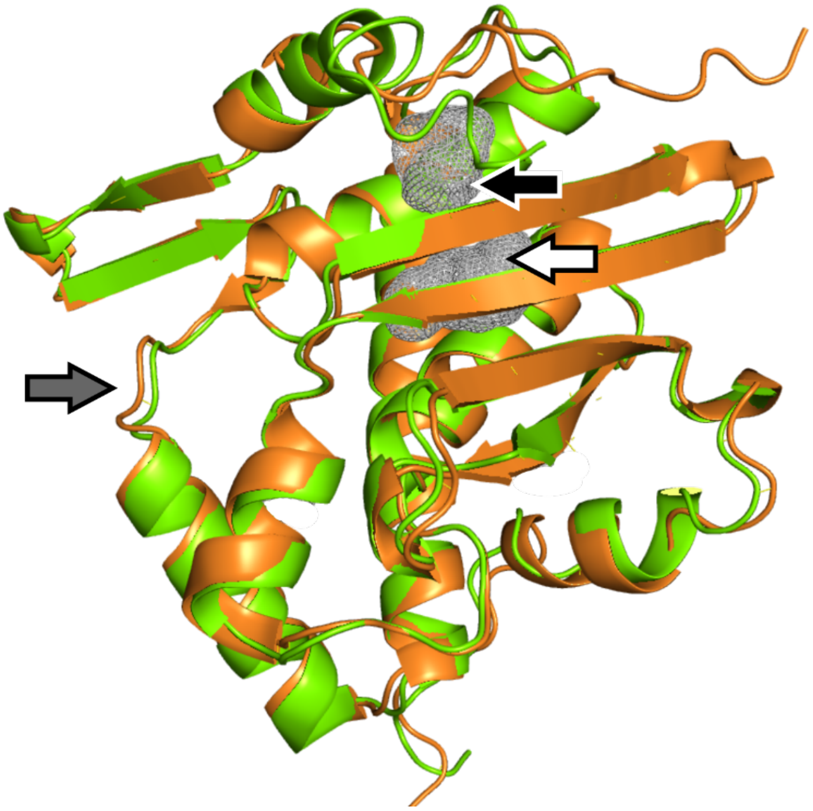

The structural model of the wild-type XylR A domain prepared previously by I-TASSER server (Dvořák et al., 2021) was aligned with a new model prepared by AlphaFold structure prediction software (orange; (Senior et al., 2020). Root mean square deviation (RMSD) of atomic positions (1,587 atoms) of aligned top-ranked models prepared by I-TASSER (in green) or AlphaFold (in orange) was 3.218, indicating high similarity between the models. The updated AlphaFold model of the A domain showed, as did the previous I-TASSER model, all typical structural features of NtrC superfamily of bacterial enhancer-binding proteins (Dvořák et al., 2021) i.e., mixed α/β fold of seven α helices and seven β strands, buried binding pocket (grey wireframe highlighted with white arrow) with tunnel-like entrance (grey wireframe highlighted with black arrow), or dimerization interface of several loops and α helices (grey arrow). The core part of the domain comprises a bundle of three α helices and a four-stranded antiparallel β sheet with a binding pocket in between.

Supplementary Figure S3. Access tunnel, binding pocket, and relevant amino acid residues that contribute to their shaping in the AlphaFold model of the wild-type XylR A domain.

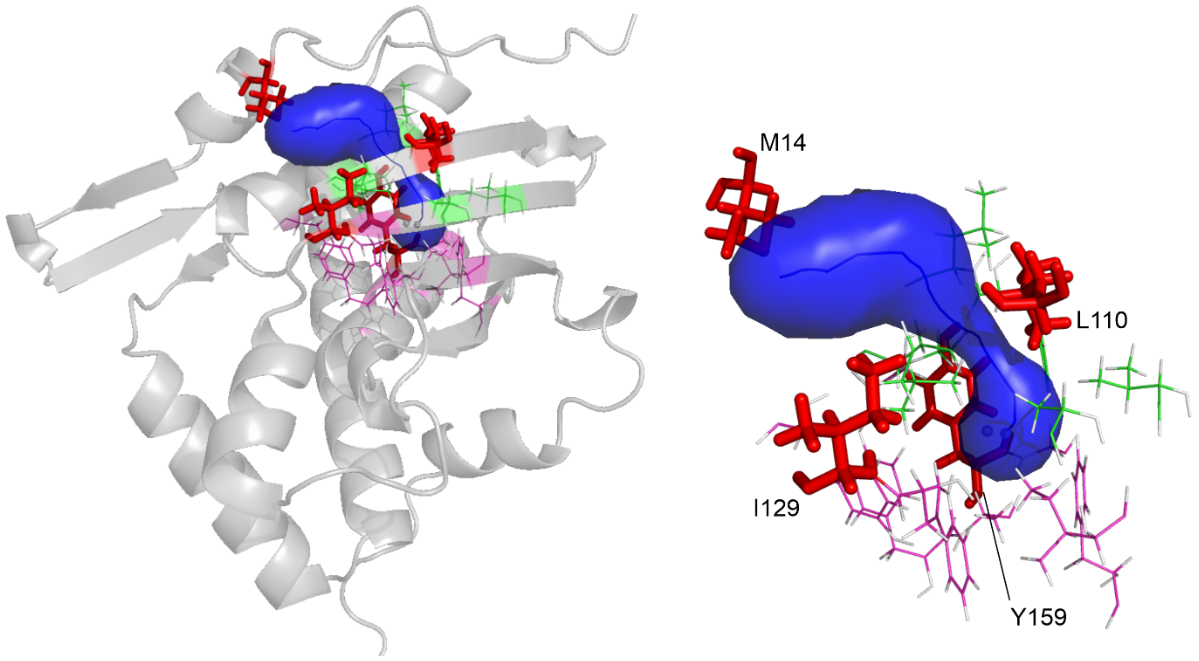

Picture on right shows the binding pocket and the entry tunnel that connects it with the bulk external solvent predicted by CAVER web 1.1 (Stourac et al., 2019) in the XylR A domain structure calculated by AlphaFold (visualization in PyMOL). Picture on right is a zoomed visualization of the tunnel, pocket and relevant surrounding amino acid residues. The tunnel and the pocket are shown as blue surface, tunnel bottleneck residues (F93, L94, P97, V108, V124, and A126) are shown as green lines. Remaining residues that form the binding pocket (G96, Y100, W128, Y155, A156, G160, F170, and I185) are shown as magenta lines. Four amino acids mutated in XylR A domain variants V18 (L110), V101 (Y159), and Va (M14 and I129) are highlighted as red sticks. M14 shapes tunnel entry, L110 belongs among tunnel bottleneck residues, and Y159 is a highly conserved binding pocket residue. I129 lies in a proximity to the conserved residues of the binding pocket.

REFERENCES

Dvořák, P., Alvarez-Carreño, C., Ciordia, S., Paradela, A., and de Lorenzo, V. (2021) An updated structural model of the A domain of the *Pseudomonas putida* XylR regulator poses an atypical interplay with aromatic effectors. *Env Microbiol* **23**: 4418-4433.

Senior, A.W., Evans, R., Jumper, J., Kirkpatrick, J., Sifre, L., Green, T. et al. (2020) Improved protein structure prediction using potentials from deep learning. *Nature* **577**: 706-710.

Stourac, J., Vavra, O., Kokkonen, P., Filipovic, J., Pinto, G., Brezovsky, J. et al. (2019) Caver Web 1.0: identification of tunnels and channels in proteins and analysis of ligand transport. *Nucleic Acids Res* **47**: W414-w422.
